# Heterogeneity of cortical pTDP-43 inclusion morphologies in amyotrophic lateral sclerosis

**DOI:** 10.1186/s40478-023-01670-2

**Published:** 2023-11-13

**Authors:** Rachel H. Tan, Heather McCann, Claire E. Shepherd, Monica Pinkerton, Srestha Mazumder, Emma M. Devenney, Gabrielle L. Adler, Dominic B. Rowe, Jillian Kril, Glenda M. Halliday, Matthew C. Kiernan

**Affiliations:** 1 Brain and Mind Centre, University of Sydney, Sydney, NSW Australia; 2 Faculty of Medicine and Health, School of Medical Sciences, University of Sydney, Camperdown, NSW Australia; 3 Institute of Clinical Neurosciences, Royal Prince Alfred Hospital, Sydney, NSW Australia; 4 Macquarie University Centre for Motor Neuron Disease Research, Faculty of Medicine, Health and Human Sciences, Macquarie University, Sydney, NSW Australia; 5 Dementia Research Centre, Macquarie Medical School, Macquarie University, Sydney, NSW Australia; 6 Neuroscience Research Australia, Randwick, NSW Australia

**Keywords:** Amyotrophic lateral sclerosis, Frontotemporal lobar degeneration, pTDP-43

## Abstract

**Background:**

Despite the presence of significant cortical pTDP-43 inclusions of heterogeneous morphologies in patients diagnosed with amyotrophic lateral sclerosis (ALS), pathological subclassification is routinely performed in the minority of patients with concomitant frontotemporal dementia (FTD).

**Objective:**

In order to improve current understanding of the presence and relevance of pathological pTDP-43 subtypes in ALS, the present study examined the pattern of cortical pTDP-43 aggregates in 61 ALS cases without FTD.

**Results:**

Based on the presence, morphology and composition of pTDP-43 pathology, three distinct ALS-TDP subtypes were delineated: (1) A predominant pattern of pTDP-43 granulofilamentous neuronal inclusions (GFNIs) and grains that were immuno-negative for p62 was identified in 18% of cases designated ALS-TDP type E; (2) neuronal cytoplasmic inclusions (NCIs) that were immuno-positive for both pTDP-43 and p62 were observed in 67% of cases assigned ALS-TDP type B; and (3) scarce cortical pTDP-43 and p62 aggregates were identified in 15% of cases coined ALS-TDP type SC (scarce cortical). Quantitative analyses revealed a significantly greater burden of pTDP-43 GFNI and grains in ALS-TDP type E. Principal component analysis demonstrated significant relationships between GFNIs, grains and ALS-TDP subtypes to support the distinction of subtypes E and B. No significant difference in age at death or disease duration was found between ALS-TDP subgroups to suggest that these subtypes represent earlier or later stages of the same disease process. Instead, a significantly higher ALS-TDP stage, indicating greater topographical spread of pTDP-43, was identified in ALS-TDP type E. Alzheimer’s disease neuropathological change (ABC score ≥ intermediate) and Lewy body disease (Braak stage ≥ IV) was more prevalent in the ALS-TDP type SC cohort, which also demonstrated a significantly lower overall cognitive score.

**Conclusion:**

In summary, the present study demonstrates that ALS-TDP does not represent a single homogenous neuropathology. We propose the subclassification of ALS-TDP into three distinct subtypes using standard immuno-stains for pTDP-43 and p62 in the motor cortex, which is routinely sampled and evaluated for diagnostic neuropathological characterisation of ALS. We propose that future studies specify both clinicopathological group and pTDP-43 subtype to advance current understanding of the pathogenesis of clinical phenotypes in pTDP-43 proteinopathies, which will have significant relevance to the development of targeted therapies for this heterogeneous disorder.

**Supplementary Information:**

The online version contains supplementary material available at 10.1186/s40478-023-01670-2.

## Introduction

Amyotrophic lateral sclerosis (ALS) is a rapidly progressive neurodegenerative disease underscored by diverse yet poorly understood pathomechanisms that contribute to differential therapeutic responses and unsuccessful drug development [1, 2]. ALS is characterised by the loss of upper and lower motor neurons with cytoplasmic pTDP-43 inclusions observed in > 90% of patients [3, 4]. Approximately 5–10% of patients with ALS also meet diagnostic criteria for frontotemporal dementia (FTD). Based on the predominant morphology and distribution of cortical pTDP-43 pathology in these FTD-ALS cases, they are routinely classified into one of four frontotemporal lobar degeneration (FTLD) pathological subtypes (FTLD-TDP types A-D) [5, 6], with a fifth subtype (FTLD-TDP type E) recently described [7]. Although pTDP-43 pathology is also present in the frontal and/or temporal cortices of ~ 70% of ALS cases without FTD [8], these ALS cases are not routinely classified into an FTLD-TDP subtype. Importantly however, pTDP-43 aggregates accumulate in the cortical neurons of the motor cortex in ALS [4, 9–11] to a similar extent as observed in FTLD [12] and heterogeneity in these cortical pTDP-43 aggregates is also recognised in ALS [13]. In order to improve current understanding of the presence and relevance of pathological pTDP-43 subtypes in ALS, the present study assessed the pattern of motor cortical pTDP-43 in 61 patients without concomitant FTD, and assessed the clinical phenotype of these pathological subtypes to identify clinicopathological associations.

## Methods

All cases with a pathological diagnosis of ALS-TDP were selected from a neuropathological series collected by the Sydney Brain Bank through regional brain donor programs in Sydney, Australia. The brain donor programs hold approval from the Human Research Ethics Committees of the University of New South Wales and comply with the statement on human experimentation issued by the National Health and Medical Research Council of Australia. Patients were diagnosed using standard clinical diagnostic criteria [1, 14–18] following a medical interview, cognitive testing [19], and informant history. Patients with cognitive or behavioural impairment that met criteria for ALS-FTD [16] were not included in this study. A total of 61 cases fulfilled these inclusion criteria. Standardized neuropathological characterization was performed [20] and all ALS cases demonstrated upper and lower motor neuron degeneration accompanied by cytoplasmic pTDP-43 inclusions in the lower motor neurons. Macroscopic atrophy in the frontal and/or temporal lobes was not observed. All cases had previously been staged for topographical progression of pTDP-43 [9] and screened for mutations in the *C9ORF72* gene [21] which was identified in 18% of these cases (n = 11). No mutations in other known ALS genes were identified in this series. An intermediate level of Alzheimer’s disease (AD) neuropathological change, which is considered adequate explanation for cognitive impairment [22] was identified in 5% of cases (n = 3) and Lewy body disease (LBD) ≥ Braak stage IV pathology, which is adequate explanation for clinical parkinsonism or cognitive impairment [22, 23] was identified in 3% of cases (n = 2). This research project was approved by the Human Research Ethics Committee of the University Sydney.

### Pathological classification and regional assessments of pTDP-43 subtypes

Pathological classification was based on pTDP-43 aggregates in the motor cortex, which is the earliest cortical region implicated in ALS [24, 25]. TDP-43 pathology accumulates in the cortical neurons of the motor cortex of ALS cases [4, 10] and demonstrates a similar pathological burden to that seen in the motor cortex of FTLD cases [12]. Both pTDP-43 and p62 immunostained sections were assessed since: 1) pTDP-positive p62-negative aggregates have been identified and associated with ALS [7, 12, 26]; 2) pTDP-negative p62-positive inclusions have been described in *C9ORF72*-carriers [27]; and 3) the classification of FTLD-TDP is based on a scheme originally developed on ubiquitinated pathology [6]. In addition to the motor cortex, the frontal cortex, which is routinely assessed for the classification of FTLD-TDP subtypes and is implicated in ALS-TDP stage 3, was also assessed to determine if pTDP-43 aggregates and subtypes differ in cortical regions implicated in early versus later stages of pTDP-43 progression. Pathological assessments were performed by two raters blind to case details with a 94% inter-observer agreement for subclassification of ALS-TDP types E and B.

### Quantitative analysis of motor cortical pTDP-43 morphologies

Ten µm thick formalin-fixed, paraffin-embedded tissue sections from each region-of-interest were immunostained with antibodies against phospho-TDP-43 (S409/410) (Cosmo Bio Co, TIP-PTD-M01, 1:80,000) and p62 (BD Biosciences, 610,833, 1:500) using a Ventana BenchMark GX autostainer. Both antibodies underwent 30 min antigen retrieval with CC1 reagent followed by a one hour primary antibody incubation and visualization using Optiview DAB IHC Detection Kit. Slides were digitally scanned using the Olympus VS-120 slidescanner at 20 × magnification. Regions of interest measuring 2000 µm × 1000 µm were defined and the percentage area occupied by granulofilamentous pTDP-43 inclusions, pTDP-43 grains and round pTDP-43 neuronal cytoplasmic (NCIs) were quantified using QuPath (version 0.3.2) (Additional file 1: Supplementary Figure 1). The severity of pTDP-43 deposition across the upper and lower cortical layers was graded on a four-point scale: 0 = no detectable pathology across the entire section; 1 = mild (some pathology observed in most fields of view); 3 = moderate; and 4 = frequent as previously described [28]. The association between pathological TDP-43 with p62 or ubiquitin was assessed using double-labelled immunofluorescence on 10 µm thick sections as previously described [29]. Briefly, primary antibodies were incubated overnight at 4 °C (anti-pTDP-43, Cosmo Bio TIP- PTD-M01; anti-ubiquitin, Merck U5379; anti-pTDP-43 rabbit polyclonal, Cosmo Bio Co TIP- PTD-P02, anti-p62, BD Biosciences, 610,833). The sections were then incubated with species-specific secondary antibodies for 2 h at room temperature (AlexaFluor 488-labelled anti-mouse IgG, Thermofisher) and Alexa 568-labelled anti-rabbit, Thermofisher), sections were counterstained with 4′,6-diamidino-2-phenylindole DAPI (Thermofisher, 62,248, 1 mg/mL) and visualised using the Leica DM6000 microscope.

### Cognitive assessment

Patients were assessed with the Addenbrooke’s Cognitive Examination (ACE-III) [19], which evaluates attention/orientation, fluency, memory, language and visuospatial function to yield a maximum score of 100.

### Statistics

Statistical analysis was performed using SPSS (Version 28) with a *p *value < 0.05 taken as significant. Group differences were determined using a one-way ANOVA followed by Bonferroni *post* hoc tests for age, disease duration, postmortem delay, density of different pTDP-43 inclusions, ALS-TDP stage and ACE-III scores, and with chi-square test for gender, site of disease onset, *C9ORF72* and co-pathologies. Any relationships between variables were assessed using principal component factor analysis (PCA), which included the density of different pTDP-43 inclusions in the motor cortex, ALS-TDP group, genetic mutation and demographic variables (age and disease duration).

## Results

### Cortical pTDP-43 subtypes in ALS (Figs. 1, 2)

**Fig. 1:**
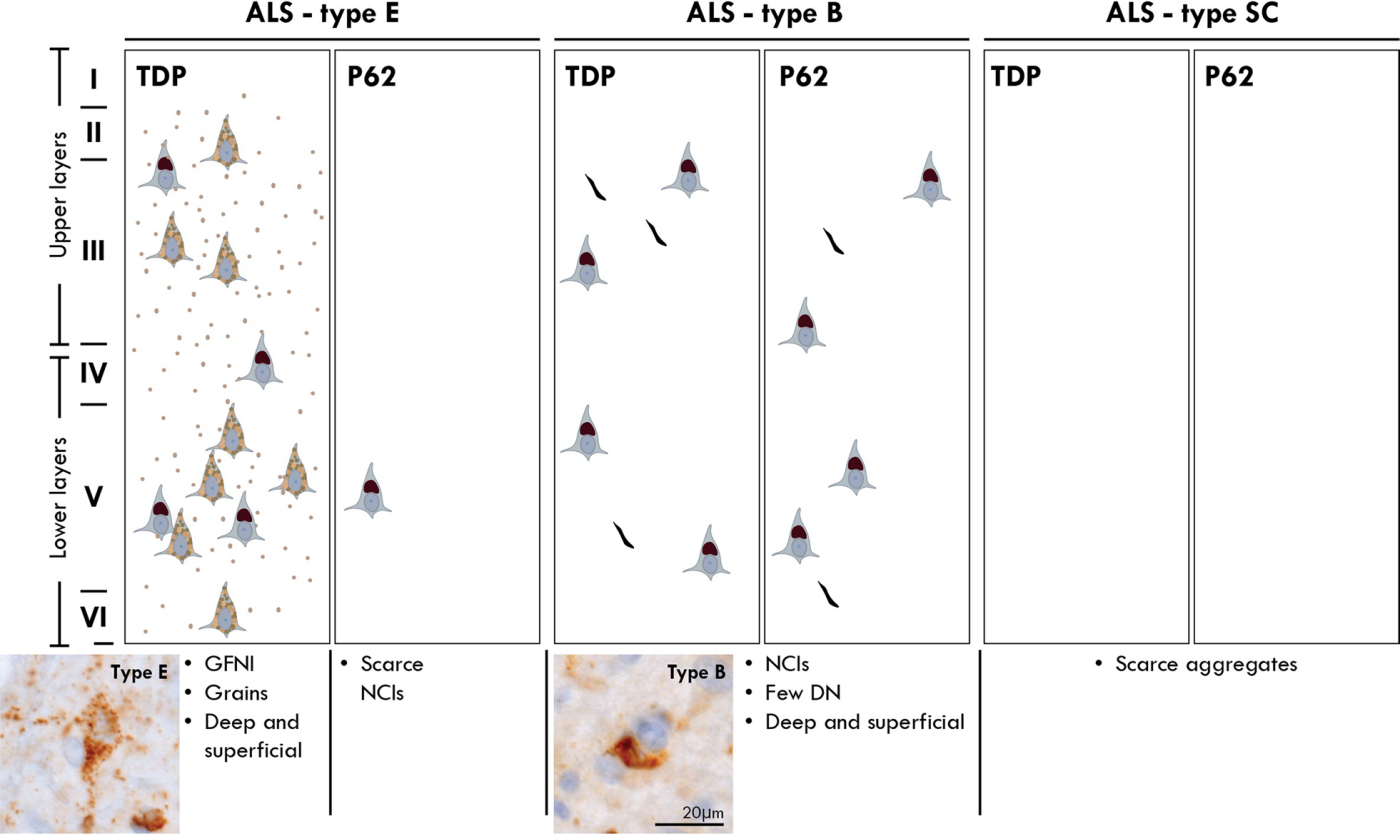
A schematic illustration of the motor cortex for the classification of ALS-TDP subtypes. Cases with a predominant pattern of pTDP-positive granulofilamentous neuronal inclusions (GFNIs) and grains not identified on corresponding p62-immunostained sections were assigned a type E; cases with round neuronal cytoplasmic inclusions ± few dystrophic neurites that were observed throughout the cortical layers of both pTDP-43 and p62 immunostained sections were assigned a type B; cases with scarce pTDP-43 and p62 aggregates that were insufficient for subclassification were assigned a type SC (scarce cortical)

**Fig. 2:**
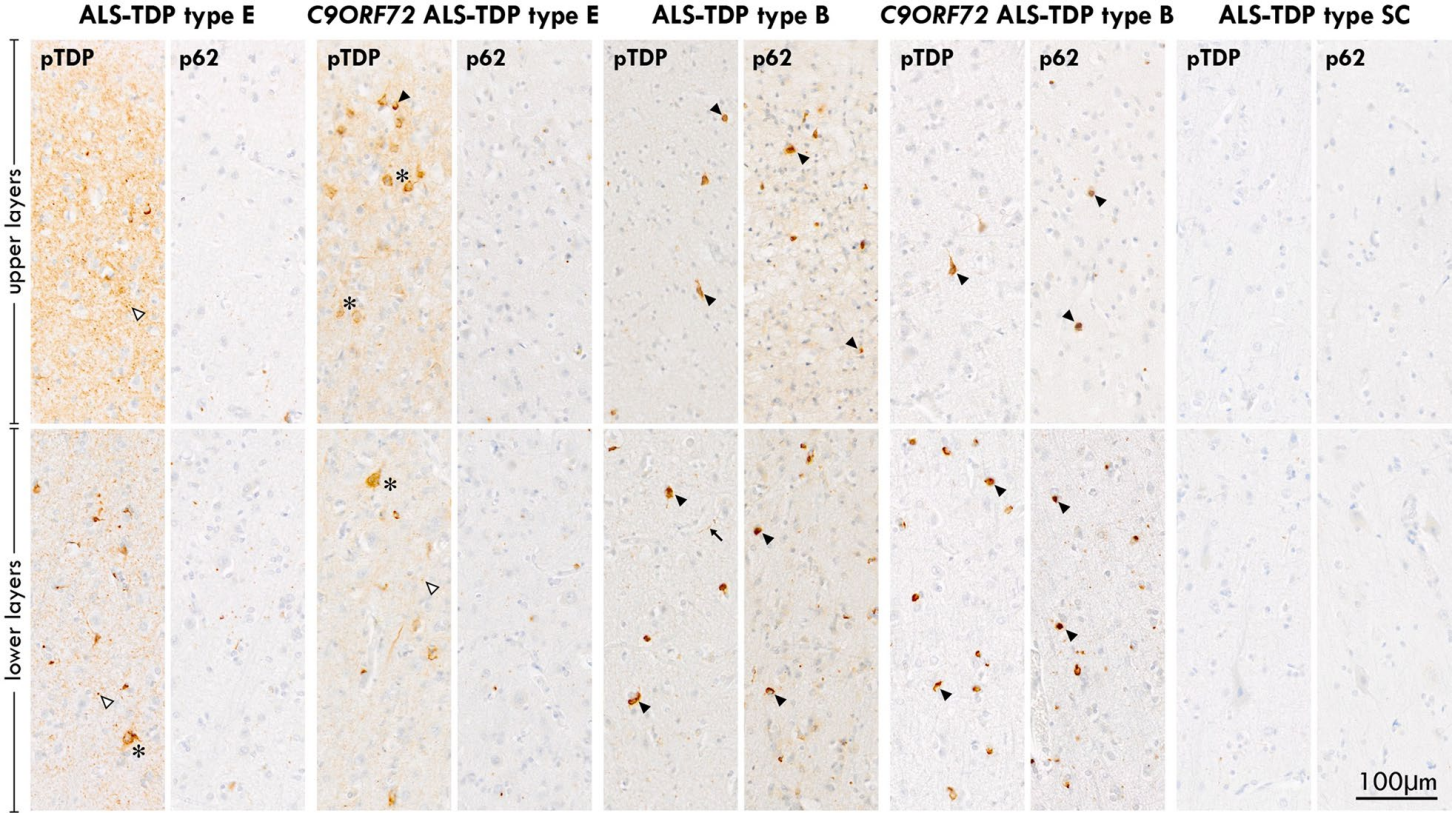
Micrographs of the characteristic pathologies observed in the motor cortex of ALS-TDP subtypes. Cases with ALS-TDP type E demonstrated a predominant pattern of pTDP-positive granulofilamentous neuronal inclusions (GFNIs, asterix) and grains (white arrowhead) not identified on corresponding p62-immunostained sections in both *C9ORF72*-carriers and non-carriers. Cases with ALS-TDP type B had a predominant pattern of round neuronal cytoplasmic inclusions (black arrowhead) and occasional dystrophic neurites (arrow) that were observed throughout the cortical layers of both pTDP-43 and p62 immunostained sections in both *C9ORF72*-carriers and non-carriers. Cases with ALS-TDP type SC (scarce cortical) had scarce pTDP-43 and p62 aggregates that could not be subtyped

#### ALS-TDP type E

Granulofilamentous neuronal inclusions (GFNI), abundant grains and oligodendroglial inclusions that were immunopositive for pTDP-43 but not p62 were identified in 18% of cases (n = 11). The morphology and composition of these aggregates resemble that recently reported in FTLD-TDP type E [7] and as such, these cases were designated ALS-TDP type E.

#### ALS-TDP type B

A predominant pattern of round neuronal cytoplasmic inclusions in superficial and deep cortical layers with relatively few dystrophic neurites were observed on both pTDP-43 and p62-immunostained sections of the motor cortex in 67% of ALS cases (n = 41). The morphology, composition and distribution of these aggregates resemble that seen in the cortical sections of FTLD-TDP type B cases and as such, these cases were designated ALS-TDP type B.

#### ALS-TDP type SC

**Fig. 3:**
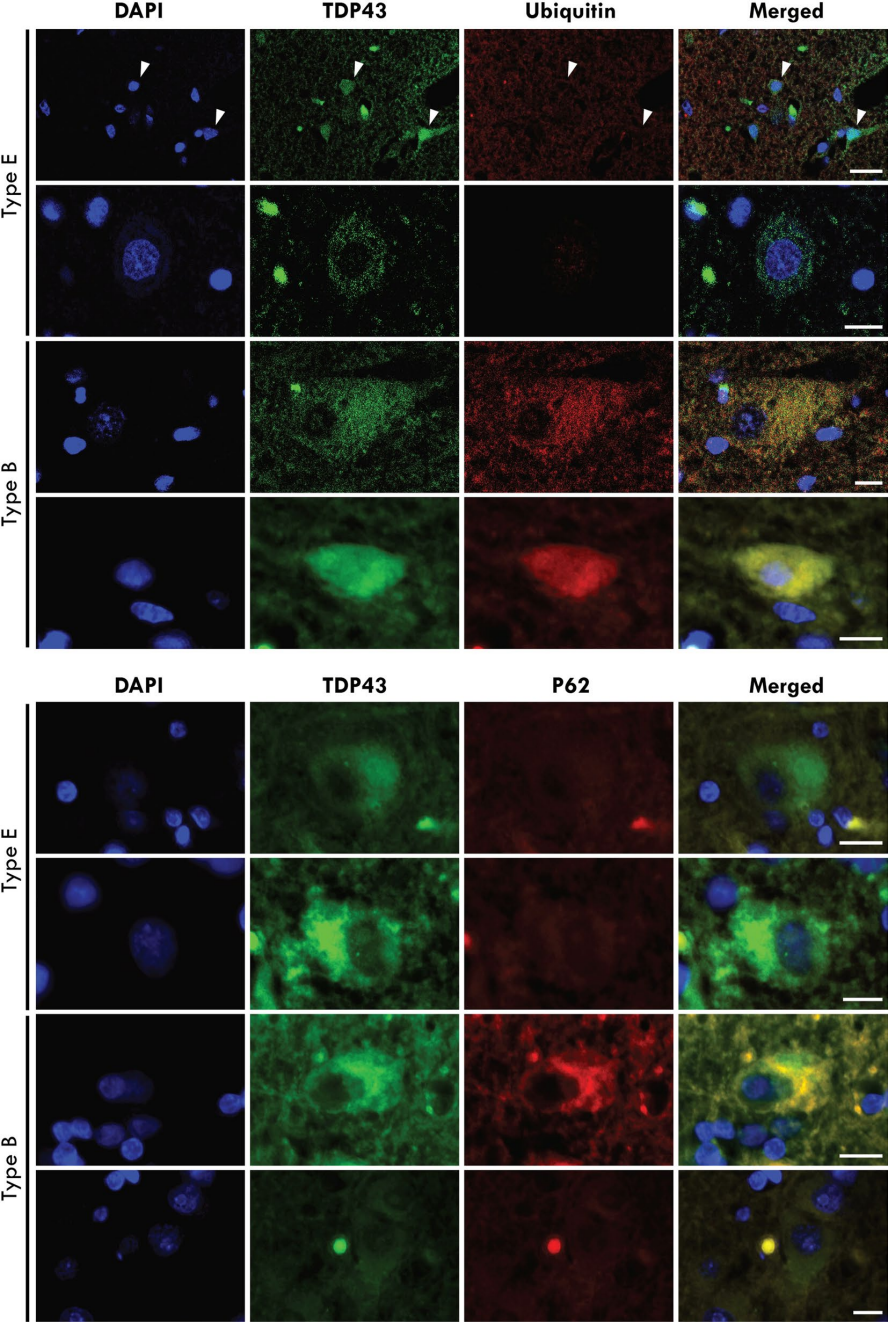
Cortical inclusions were immunopositive for pTDP-43 but not ubiquitin or p62 in ALS-TDP type E. In contrast to pTDP-43 aggregates that were ubiquitin-negative in ALS-TDP type E, pTDP-43 aggregates were ubiquitin-positive in ALS-TDP type B cases. Similarly, pTDP-43 aggregates were p62-negative in ALS-TDP type E but p62-positive in ALS-TDP type B cases

Scarce pTDP-43 and p62 aggregates were observed in the motor cortex of 15% of cases (n = 9) and were designated ALS-TDP type SC (scarce cortical). Double-labelled immunofluorescence confirmed the absence of ubiquitin and p62 immunoreactivity in the pTDP-43 aggregates of ALS-TDP type E cases (Fig. 3). In contrast, the pTDP-43 aggregates in ALS-TDP type B cases demonstrated colocalization with p62 and ubiquitin (Fig. 3).

### Quantitative analyses of pTDP-43 inclusion morphologies

**Fig. 4:**
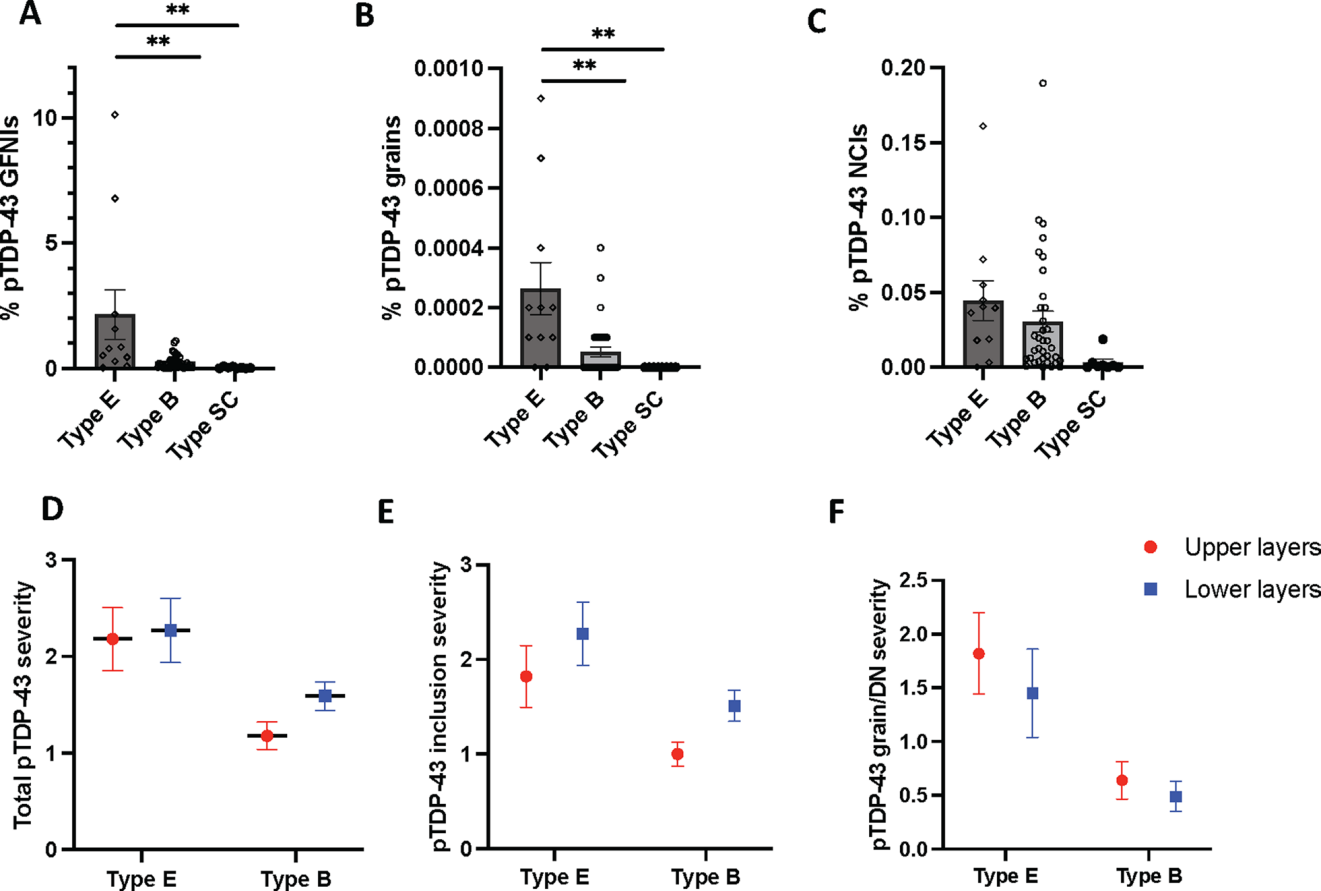
Quantitative analyses of motor cortical pTDP-43 morphologies in ALS-TDP subgroups. A significantly greater burden of pTDP-43 granulofilamentous neuronal inclusions (GFNIs) and grains were found in ALS-TDP type ** E** (n = 11) compared to ALS-TDP type ** B** (n = 35) and ALS-TDP type SC (n = 8) cases, even when when the two ALS-TDP type **E** cases with the most severe pathology were excluded from analysis (*p* ≤ 0.005) (** A**-** B**). No significant difference in pTDP-43 neuronal cytoplasmic inclusions (NCIs) were found between groups (** C**). Semi-quantitative analysis of pTDP-43 burden revealed a similar burden of pathological pTDP-43 in the superficial and deep layers within subtypes (** D**-** F**). **p ≤ 0.005

Assessment of motor cortical burden of pTDP-43 morphologies revealed a significantly greater burden of pTDP-43 GFNI and grains in ALS-TDP type E compared to ALS-TDP type B and ALS-TDP type SC cases (F(2,37) > 6.2, *p* ≤ 0.005; Fig. 4). No significant difference in pTDP-43 NCIs were found between groups (F(2,37) = 2.3, *p* = 0.113). Principal component factor analysis was used to assess all relationships between variables across all cases and loading scores > 0.6 was considered significant. Two separate factors accounted for > 57% of variance. Significant relationships were identified between ALS-TDP subtype (0.74 loading), % GFNI (0.86 loading) and % pTDP-43 grains (0.83 loading) (factor 1, 35% of the variance), supporting the construct that pTDP-43 GFNI and grains differentiated the ALS-TDP groups. Factor 2 accounted for 22% of the variance and showed that % round pTDP-43 NCIs (0.633 loading) associated with age (-0.63 loading), disease duration (-0.64 loading) and presence of a *C9ORF72* mutation (0.69 loading). This is consistent with the shorter disease duration, younger age at death and round NCIs characteristic of type A/B described in *C9ORF72* cases [30].

### Demographic and pathological comparisons between ALS-TDP subtypes

Group comparisons revealed no significant differences in age at death, age at disease onset, disease duration or post-mortem interval between groups (Table 1). No significant difference in the prevalence of a *C9ORF72* expansion was identified between groups (*X*(2) = 2.41, *p* = 0.3) (Table 1 and Fig. 2). A significantly greater mean ALS-TDP stage was found in ALS-TDP type E compared to ALS-TDP type B and ALS-TDP SC cases (F(2, 58) = 6.569, *p* = 0.003, Table 1). Intermediate Alzheimer’s disease (AD) neuropathological change and LBD ≥ Braak stage IV pathology was more prevalent in the ALS-TDP SC cohort compared to the ALS-TDP type B and ALS-TDP type E cohorts (*X*(2) = 14.49, *p* < 0001, Table 1). Within the ALS-TDP type B cohort, no significant difference in age at death, disease duration or post-mortem interval (PMI) was found between *C9ORF72*-carriers and sporadic cases (age at death (mean ± SD): 59 ± 8 years in *C9ORF72* cases, 66 ± 11 years in sporadic cases, *p* = 0.1; disease duration (mean ± SD): 2 ± 1 years in *C9ORF72* cases, 3 ± 2 years in sporadic cases, *p* = 0.2; PMI (mean ± SD): 24 ± 10 h in *C9ORF72* cases, 28 ± 15 h in sporadic cases, *p* = 0.5). However, a significantly higher ALS-TDP stage was identified in *C9ORF72*-carriers compared to sporadic ALS cases (Mean ± SD ALS-TDP stage: 3.9 ± 0.3 in *C9ORF72*-carriers, 2.3 ± 1.1 in sporadic ALS cases, *p* < 0.001). ALS-TDP stage was similar in ALS-TDP type E cases with and without a *C9ORF72* expansion (Mean ± SD ALS-TDP stage: 4.0 ± 0.0 in *C9ORF72*-carriers, 3.9 ± 0.3 in sporadic ALS cases).

**Table 1:** Demographic, clinical and pathological characteristics of ALS-TDP subtypes

	ALS-TDP type B	ALS-TDP type E	ALS-TDP type SC	*P* value
N (% male)	41 (56%)	11 (36%)	9 (78%)	0.2
Age at death (years)	64 ± 11	65 ± 7	68 ± 12	0.6
Age at disease onset (years)	62 ± 11	63 ± 7	64 ± 12	0.8
Disease duration (years)	3 ± 1	3 ± 2	4 ± 2	0.2
Site of onset UL/LL/Bulbar (n)	15/11/15	6/2/3	4/4/1	0.5
Postmortem interval (hours)	27 ± 14	27 ± 14	29 ± 21	0.9
*C9ORF72*% (n)	22% (9)	18% (2)	0% (0)	0.3
Mean ALS-TDP stage	2.6 ± 1.2	3.9 ± 0.3*	2.2 ± 1.5	0.003
Intermediate AD or LBD Braak stage IV-VI (%)	5%	0%	45%*	0.001
ACE assessment % (n)	46% (19)	82% (9)	44% (4)^#^	0.3
ACE-III total /100	89 ± 10	92 ± 6	76 ± 16*	0.03
Time between cognitive assessment and death (years	1.4 ± 1.3	1.4 ± 0.7	2.8 ± 1.9	0.1

Data is presented as mean ± standard deviation or prevalence. *UL* upper limb; *LL* lower limb; *LBD* lewy body disease; *ADNC* Alzheimer’s disease neuropathological changes; *ACE-III* Addenbrooke’s Cognitive Examination third edition [19]. ^#^Despite the absence of cognitive scores for 75% of ALS-TDP type SC case with intermediate AD or neocortical LBD (n = 3), a significantly lower overall cognitive performance was identified in this subgroup. *p < 0.05 compared to the two other pathological groups

### Regional inclusion morphology and pathological subtype

**Fig. 5:**
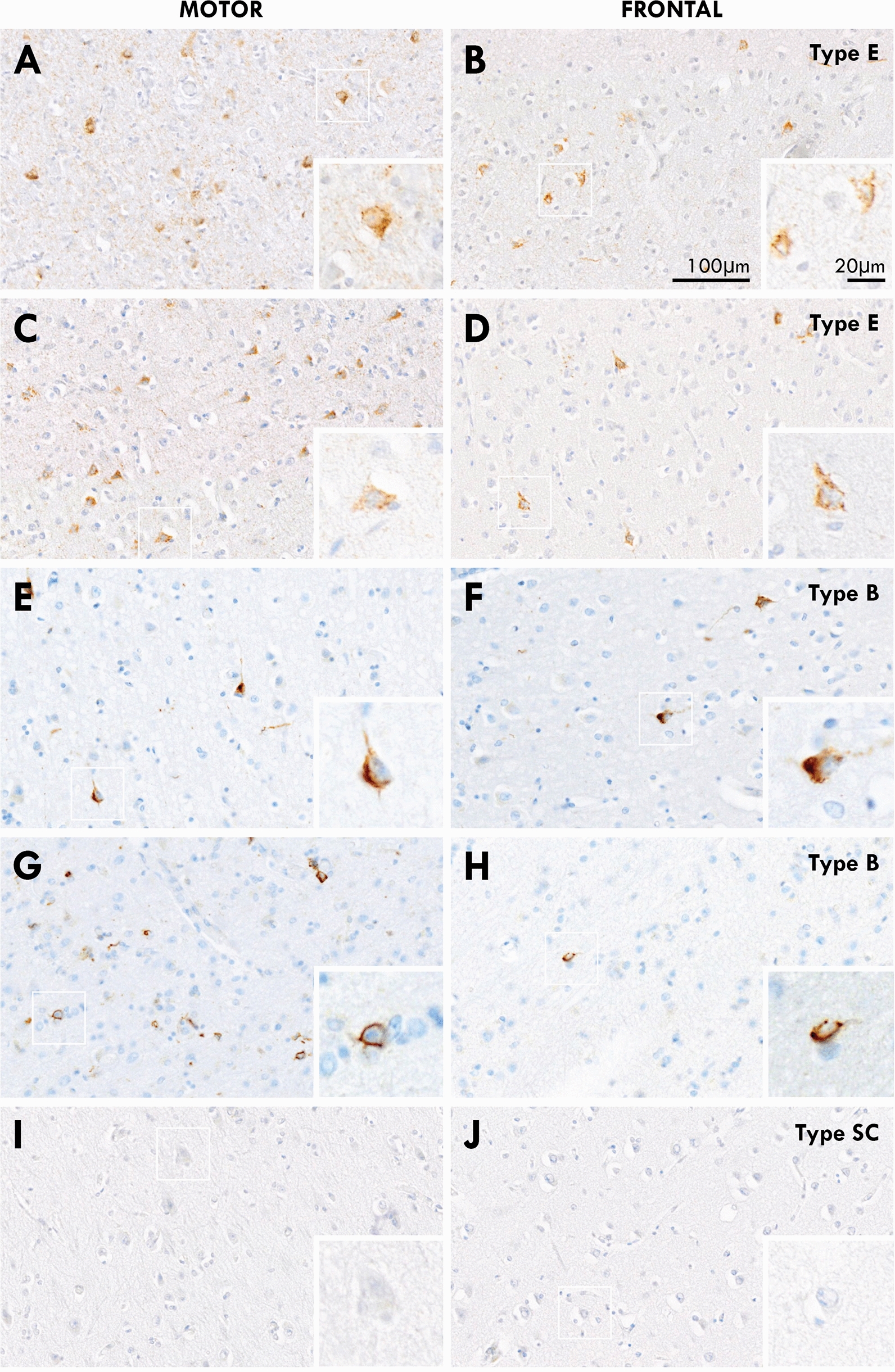
A side-by-side comparison of pTDP-43 in the motor and frontal cortex of the same patients with ALS-TDP type E (**A**-**D**), ALS-TDP type B (**E**- **H**) and ALS-TDP type SC (**I**, **J**). Regional differences in pathological subtypes were not identified within cases

**Fig. 6:**
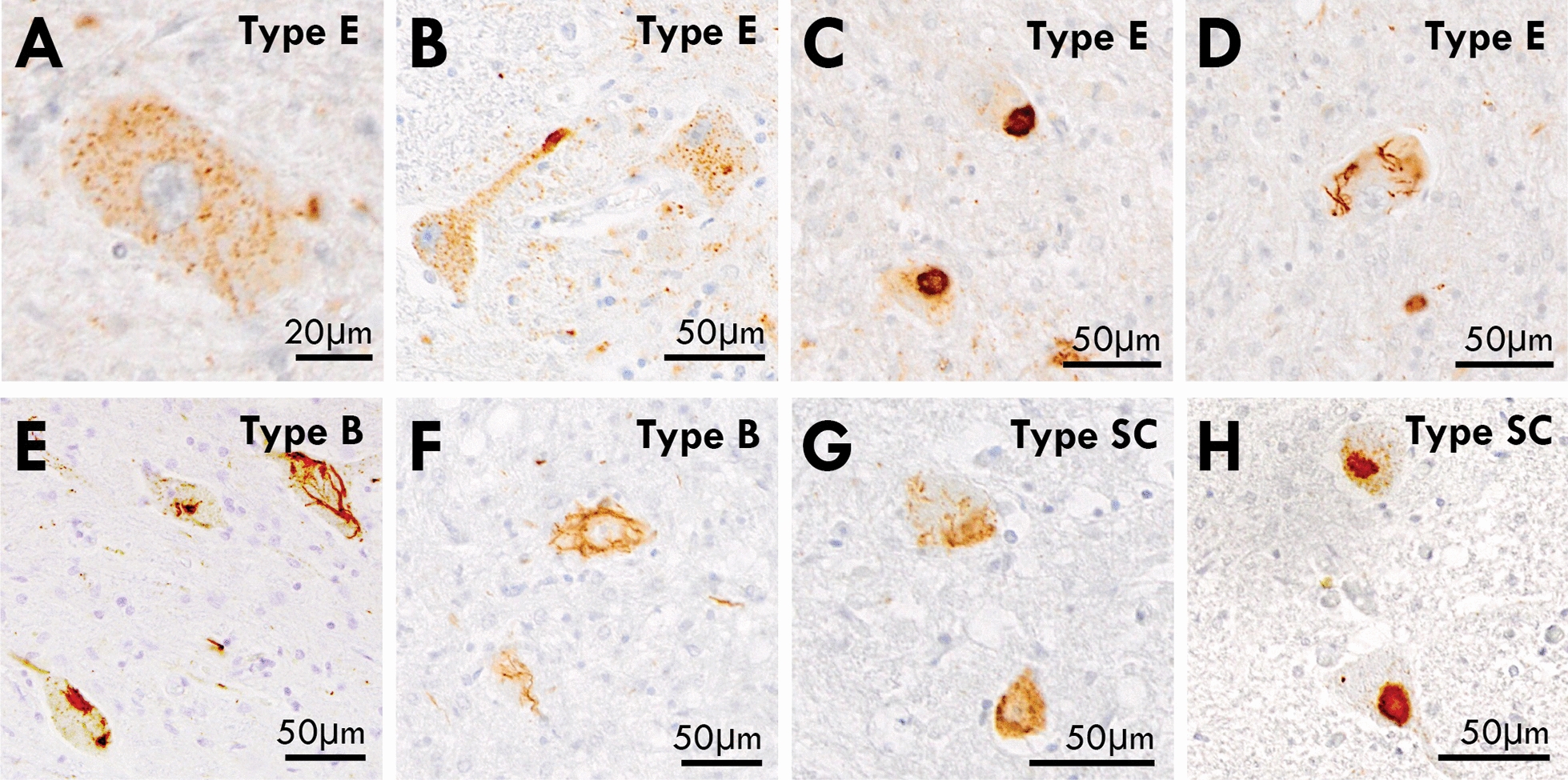
pTDP-43 inclusions in the spinal cord of ALS-TDP subtypes. **A** predominance of granulofilamentous neuronal inclusions and grains were observed in the anterior horn of the spinal cord of ALS-TDP type **E** cases (**A**, **B**) and these were accompanied by occasional dense compact and skein-like inclusions (**C**, **D**) similar to that observed in ALS-TDP type **B** (**E**, **F**) and ALS-TDP type SC cases (**G**, **H**)

To determine if the GFNIs characteristic of ALS-TDP type E represent immature pTDP-43 inclusions that have yet to coalesce into the more prevalent ALS-TDP type B morphology which is ubiquitinated, the frontal cortex, which is affected later in pathological progression (and consequently would demonstrate comparatively “earlier” pathology by the end of the disease progression) were assessed. ALS-TDP type B cases with pTDP-43 pathology in the frontal cortices (n = 21) demonstrated similar round neuronal cytoplasmic inclusions with few neurites that were immunopositive for p62 in these “later affected” regions. As expected, all ALS-TDP type E cases demonstrated GFNI and grains in the frontal cortex that were consistent with that seen in the motor cortex of these cases. Regional differences in pathological subtypes were not identified within cases here nor have they been previously reported (Fig. 5). In the spinal cord, a predominance of pTDP-43 GFNIs, grains and oligodendroglial inclusions were observed in ALS-TDP type E cases (Fig. 6). These inclusion morphologies were accompanied by occasional dense compact and skein-like pTDP-43 inclusions, similar to that observed in ALS-TDP type B and type SC (Fig. 6).

### Cognitive profiles

ACE-III scores [19] were available for 82% of the ALS-TDP type E cohort (n = 9, including the n = 2 with a *C9ORF72* expansion), 46% of the ALS-TDP type B cohort (n = 19, including n = 5 with a *C9ORF72* expansion) and 44% of the ALS-TDP type SC cohort (n = 4, including the n = 1 case with LBD stage IV), with no significant group differences in the years between assessments and death (Table 1). Despite the absence of cognitive scores for the ALS-TDP type SC case with neocortical LBD (Braak stage VI) (n = 1) and intermediate AD neuropathological changes (n = 2), a significantly lower overall cognitive performance was identified in ALS-TDP type SC compared to ALS-TDP type E and ALS-TDP type B cases (*p* < 0.05) (Table 1). Within the ALS-TDP type B cohort, no significant difference in ACE scores were identified between *C9ORF72*-carriers and non-carriers (Mean ± SD: 94 ± 2 in *C9ORF72*-carriers, 88 ± 11 in non-carriers, *p* = 0.3).

## Discussion

The present study examined and reports, for the first time, three distinct histological subtypes in ALS cases without FTD: (1) In 18% of cases, a predominant pattern of pTDP GFNIs and grains, which were immuno-negative for p62 were identified, and these cases were designated ALS-TDP type E; (2) In 67% of cases, a predominant pattern of round NCIs that were immuno-positive for both pTDP-43 and p62 were observed, and these cases were designated ALS-TDP type B; (3) In 15% of cases, scarce cortical pTDP-43 and p62 aggregates were observed, and these cases were coined ALS-TDP type SC (scarce cortical).

Quantitative analyses identified a significantly greater burden of pTDP-43 GFNIs and grains in ALS-TDP type E. PCA analysis of pTDP-43 inclusion morphologies revealed significant associations between GFNIs, grains and ALS-TDP subtypes to support that these pathologies segregate with ALS-TDP type E. No significant difference in age of disease onset, death or survival was found between groups to suggest that these subtypes represent earlier or later-stage disease pathologies. Further to this, pTDP-43 morphology and pattern of p62-immunoreactivity in each case was consistent across early- and late-affected cortical regions, indicating that non-ubiquitinated GFNIs are not a predecessor to ubiquitinated NCIs. Instead, a significantly higher ALS-TDP stage was identified in the ALS-TDP type E cohort. Interestingly, a lower overall cognitive score and higher incidence of Alzheimer’s disease neuropathological change (≥ intermediate level) or LBD (Braak stage ≥ IV) was identified in the ALS-TDP SC cohort. To date, pathological subclassification of pTDP-43 subtypes is routinely performed in FTLD, as demonstrated by the nomenclature “FTLD-TDP subtypes A-E”. Given the similarities in the pattern of cortical pTDP-43 in two of our ALS subgroups with that identified in the FTLD-TDP subtypes, we have adopted the same alphabetised subclassification and propose that future studies consider specifying clinicopathological group followed by pTDP-43 subtype (eg. FTLD-ALS type E) to advance pathomechanistic understanding of clinical phenotypes in pTDP-43 proteinopathies.

Widespread pTDP-positive p62-negative GFNI, grains and oligodendroglial inclusions were first described in seven FTD cases with motor neuronal pTDP-43 and rapid disease durations of one to three years [7]. This pathology, coined FTLD-TDP type E, bore a striking resemblance to the cortical pTDP-43 identified in patients with FTLD-ALS [12], sporadic ALS [13] and primary progressive aphasia with ALS [26], suggesting that the rapidity of these seven FTLD-TDP type E cases was due to the concomitant presence of ALS [31]. However, most of these studies had not assessed pTDP-43 aggregates for p62-immunoreactivity and as such, whether Type E represented a distinct subtype, or “early stage” Type B, remained a topic of contention [32]. The present study in 61 ALS cases corroborates the existence of an obvious disparity in cortical pTDP-43 morphology and p62-immunoreactivity in a subset of cases at end-stage. These cases, assigned the nomenclature ALS-TDP type E, demonstrated no significant difference in disease duration or age at death compared to cases with the more prevalent ALS-TDP type B pathology to suggest that one represents an earlier cross-sectional process in pathological development of the other. Instead, the significantly higher ALS-TDP stage of type E cases here converges with the distinct biochemical, seeding and spreading properties associated with this pathology [7, 32, 33] to suggest that this is a unique pTDP-43 strain that may have been overlooked in previous neuropathological series that only included cases with significant neocortical ubiquitin-immunoreactive pathology [34].

In contrast to a previous study that assessed the inclusion morphologies in the ~ 30% of ALS cases with pTDP-43 in the hippocampus [13], this study assessed the earliest cortical region implicated in ALS and demonstrates heterogeneous pTDP-43 inclusion morphologies in the motor cortex. We propose a classification scheme comparable to that available for FTLD [32] and that can be used independently or in conjunction with staging schemes of the topographical progression of pTDP-43 [8]. Importantly distinct structural and biochemical properties of pTDP-43 have been identified in different FTLD-TDP subtypes [33, 35, 36], underscoring the importance of an ALS-TDP classification scheme that also accounts for inclusion morphology. Consistent with previous reports in the subcortical and lower motor neurons of FTLD-TDP type E [7] and ALS-TDP type 2b cases [13], pTDP-43 GFNIs, grains, oligodendroglial inclusions accompanied by occasional compact or skein-like pTDP-43 inclusions were identified in ALS-TDP type E cases here. Although regional differences in pathological subtypes were not identified, future studies in a larger series of cortical and subcortical regions will be able to determine if morphological stages of pTDP-43 occur with disease progression within and/or across pathological subtypes.

The *C9ORF72* expansion was identified in ALS-TDP subtypes B and E but not subtype SC here. Although this may be considered basis for support of a B-E continuum, it is important to note that in FTLD, the *C9ORF72* expansion is predominantly associated with subtypes A and B [30, 37–39]. Despite the considerable overlap in pathological and biochemical properties of these two subtypes which demonstrate poor inter-rater and -laboratory agreement in their differentiation, subtypes A and B are not considered to represent a continuum [32, 33, 40]. Further to this, the *C9ORF72* expansion has also been associated with FTLD-TDP subtypes A + B and E [7, 30], highlighting the significant pathological variability associated with this expansion. Interestingly, a significantly higher ALS-TDP stage was identified in ALS-TDP type E and *C9ORF72*-type B cases, indicating greater topographical spread of pTDP-43 to extramotor regions. Perhaps surprisingly, this was not associated with lower overall cognitive scores in either of these cohorts although it is worth noting that cognitive assessments were performed midway in the disease process. Importantly however, ALS-TDP stage is an indicator of regional presence, not severity [9], and this absence of significant cognitive impairment is consistent with the lower extramotor pTDP-43 burden identified in ALS compared to FTLD cases [28, 41]. Interestingly, a lower overall cognitive score was identified in the ALS-TDP type SC cohort, which had a higher incidence of co-pathologies compared to other groups. The main limitation to this finding is the relatively small numbers of ALS-TDP type SC cases with cognitive assessments. However, given that this lower overall cognitive score was not driven by ALS-TDP type SC cases with intermediate ADNC or neocortical LBD due to the unavailability of cognitive testing in these cases with co-pathologies that have been recognised as sufficient explanation for dementia [22], it is unlikely that these findings of represent an artifact. Nevertheless, these results highlight the need for future studies in multi-centre neuropathological series of ALS cases with detailed cognitive assessments closer to end-stage to determine the prevalence of cognitive impairment and potential associations with co-pathologies in patients with scarce cortical pathology.

## Conclusions

In summary, the present study delineates three distinct histological subtypes in ALS-TDP cases according to the presence, morphology and composition of cortical pTDP-43 pathology. These pathological subtypes demonstrate no significant difference in age at death or disease duration to suggest that they capture an earlier or later cross-sectional process in pathological pTDP-43 development. Rather, they are associated with different motor cortical burden of GFNIs and grains, topographical spreads, overall cognitive performances and co-pathologies to suggest the involvement of divergent pathomechanisms in ALS-TDP. Given the similarities between two of these ALS-TDP subtypes with that seen in FTLD-TDP, we have adopted the same alphabetised classification and highlight the need for further research to elucidate whether these similar subtypes identified in different clinical phenotypes share common disease pathways. As such, we propose that future studies specify both clinicopathological phenotype and pTDP-43 subtype, which will be important for the advancement of current understanding of disease pathogeneses and the development of targeted therapies for pTDP-43 proteinopathies.

### Supplementary Information

**Additional file 1:** Supplementary Figure 1.

## Data Availability

Anonymised datasets generated during this study are available from the corresponding author on reasonable request.

## Abbreviations

ALS: Amyotrophic lateral sclerosis
FTLD: Frontotemporal lobar degeneration
FTD: Frontotemporal dementia
pTDP-43: Phosphorylated TAR DNA-binding protein 43
GFNIs: Granulofilamentous neuronal inclusions
NCIs: Neuronal cytoplasmic inclusions
SC: Scarce cortical
AD: Alzheimer’s disease
LBD: Lewy body disease
PCA: Principal component analysis

## References

[1] Kiernan MC, Vucic S, Cheah BC, Turner MR, Eisen A, Hardiman O (2011). Amyotrophic lateral sclerosis. Lancet.

[2] Kiernan MC, Vucic S, Talbot K, McDermott CJ, Hardiman O, Shefner JM (2021). Improving clinical trial outcomes in amyotrophic lateral sclerosis. Nat Rev Neurol.

[3] Tan RH, Ke YD, Ittner LM, Halliday GM (2017). ALS/FTLD: experimental models and reality. Acta Neuropathol.

[4] Braak H, Ludolph AC, Neumann M, Ravits J, Del Tredici K (2017). Pathological TDP-43 changes in Betz cells differ from those in bulbar and spinal alpha-motoneurons in sporadic amyotrophic lateral sclerosis. Acta Neuropathol.

[5] Mackenzie IR, Neumann M, Baborie A, Sampathu DM, Du Plessis D, Jaros E (2011). A harmonized classification system for FTLD-TDP pathology. Acta Neuropathol.

[6] Sampathu DM, Neumann M, Kwong LK, Chou TT, Micsenyi M, Truax A (2006). Pathological heterogeneity of frontotemporal lobar degeneration with ubiquitin-positive inclusions delineated by ubiquitin immunohistochemistry and novel monoclonal antibodies. Am J Pathol.

[7] Lee EB, Porta S, Michael Baer G, Xu Y, Suh E, Kwong LK (2017). Expansion of the classification of FTLD-TDP: distinct pathology associated with rapidly progressive frontotemporal degeneration. Acta Neuropathol.

[8] Brettschneider J, Arai K, Del Tredici K, Toledo JB, Robinson JL, Lee EB (2014). TDP-43 pathology and neuronal loss in amyotrophic lateral sclerosis spinal cord. Acta Neuropathol.

[9] Brettschneider J, Del Tredici K, Toledo JB, Robinson JL, Irwin DJ, Grossman M (2013). Stages of pTDP-43 pathology in amyotrophic lateral sclerosis. Ann Neurol.

[10] Nolan M, Scott C, Gamarallage MP, Lunn D, Carpenter K, McDonough E (2020). Quantitative patterns of motor cortex proteinopathy across ALS genotypes. Acta Neuropathol Commun.

[11] Kassubek J, Muller HP, Del Tredici K, Brettschneider J, Pinkhardt EH, Lule D (2014). Diffusion tensor imaging analysis of sequential spreading of disease in amyotrophic lateral sclerosis confirms patterns of TDP-43 pathology. Brain J Neurol.

[12] Tan RH, Yang Y, Kim WS, Dobson-Stone C, Kwok JB, Kiernan MC (2017). Distinct TDP-43 inclusion morphologies in frontotemporal lobar degeneration with and without amyotrophic lateral sclerosis. Acta Neuropathol Commun.

[13] Takeuchi R, Tada M, Shiga A, Toyoshima Y, Konno T, Sato T (2016). Heterogeneity of cerebral TDP-43 pathology in sporadic amyotrophic lateral sclerosis: evidence for clinico-pathologic subtypes. Acta Neuropathol Commun.

[14] Al-Chalabi A, Hardiman O, Kiernan MC, Chio A, Rix-Brooks B, van den Berg LH (2016). Amyotrophic lateral sclerosis: moving towards a new classification system. Lancet Neurol.

[15] Hardiman O, van den Berg LH, Kiernan MC (2011). Clinical diagnosis and management of amyotrophic lateral sclerosis. Nat Rev Neurol.

[16] Rascovsky K, Hodges JR, Knopman D, Mendez MF, Kramer JH, Neuhaus J (2011). Sensitivity of revised diagnostic criteria for the behavioural variant of frontotemporal dementia. Brain: J Neurol.

[17] Shefner JM, Al-Chalabi A, Baker MR, Cui LY, de Carvalho M, Eisen A (2020). A proposal for new diagnostic criteria for ALS. Clin Neurophysiol: Off J Int Fed Clin Neurophysiol.

[18] Strong MJ, Abrahams S, Goldstein LH, Woolley S, McLaughlin P, Snowden J (2017). Amyotrophic lateral sclerosis—frontotemporal spectrum disorder (ALS-FTSD): revised diagnostic criteria. Amyotroph Lateral Scler Frontotemporal Degener.

[19] Hsieh S, Schubert S, Hoon C, Mioshi E, Hodges JR (2013). Validation of the Addenbrooke's cognitive examination III in frontotemporal dementia and Alzheimer's disease. Dement Geriatr Cogn Disord.

[20] Brooks BR, Miller RG, Swash M, Munsat TL (2000). El Escorial revisited: revised criteria for the diagnosis of amyotrophic lateral sclerosis. Amyotroph Lateral Scler Other Motor Neuron Disord.

[21] Renton AE, Majounie E, Waite A, Simon-Sanchez J, Rollinson S, Gibbs JR (2011). A hexanucleotide repeat expansion in C9ORF72 is the cause of chromosome 9p21-linked ALS-FTD. Neuron.

[22] Montine TJ, Phelps CH, Beach TG, Bigio EH, Cairns NJ, Dickson DW (2012). National institute on aging-Alzheimer's association guidelines for the neuropathologic assessment of Alzheimer's disease: a practical approach. Acta Neuropathol.

[23] Halliday GM, Holton JL, Revesz T, Dickson DW (2011). Neuropathology underlying clinical variability in patients with synucleinopathies. Acta Neuropathol.

[24] Brettschneider J, Del Tredici K, Irwin DJ, Grossman M, Robinson JL, Toledo JB (2014). Sequential distribution of pTDP-43 pathology in behavioral variant frontotemporal dementia (bvFTD). Acta Neuropathol.

[25] Young AL, Vogel JW, Robinson JL, McMillan CT, Ossenkoppele R, Wolk DA, Hansson O (2023). Data-driven neuropathological staging and subtyping of TDP-43 proteinopathies. Brain: J Neurol.

[26] Tan RH, Guennewig B, Dobson-Stone C, Kwok JBJ, Kril JJ, Kiernan MC (2019). The under acknowledged PPA-ALS: a unique clinicopathologic subtype with strong heritability. Neurology.

[27] Al-Sarraj S, King A, Troakes C, Smith B, Maekawa S, Bodi I (2011). p62 positive, TDP-43 negative, neuronal cytoplasmic and intranuclear inclusions in the cerebellum and hippocampus define the pathology of C9orf72-linked FTLD and MND/ALS. Acta Neuropathol.

[28] Tan RH, Kril JJ, Fatima M, McGeachie A, McCann H, Shepherd C, Halliday GM (2015). TDP-43 proteinopathies: pathological identification of brain regions differentiating clinical phenotypes. Brain: J Neurol.

[29] Yang Y, Rowe D, McCann H, Shepherd CE, Kril JJ, Kiernan MC (2023). Treatment with the copper compound CuATSM has no significant effect on motor neuronal pathology in patients with ALS. Neuropathol Appl Neurobiol.

[30] Mackenzie IR, Neumann M (2020). Subcortical TDP-43 pathology patterns validate cortical FTLD-TDP subtypes and demonstrate unique aspects of C9orf72 mutation cases. Acta Neuropathol.

[31] Halliday GM, Kiernan MC, Kril JJ, Mito R, Masuda-Suzukake M, Hasegawa M (2016). TDP-43 in the hypoglossal nucleus identifies amyotrophic lateral sclerosis in behavioral variant frontotemporal dementia. J Neurol Sci.

[32] Neumann M, Lee EB, Mackenzie IR (2021). Frontotemporal lobar degeneration TDP-43-immunoreactive pathological subtypes: clinical and mechanistic significance. Adv Exp Med Biol.

[33] Porta S, Xu Y, Lehr T, Zhang B, Meymand E, Olufemi M (2021). Distinct brain-derived TDP-43 strains from FTLD-TDP subtypes induce diverse morphological TDP-43 aggregates and spreading patterns in vitro and in vivo. Neuropathol Appl Neurobiol.

[34] Mackenzie IR, Neumann M (2017). Reappraisal of TDP-43 pathology in FTLD-U subtypes. Acta Neuropathol.

[35] Arseni D, Chen R, Murzin AG, Peak-Chew SY, Garringer HJ, Newell KL, Ryskeldi-Falcon B (2023). TDP-43 forms amyloid filaments with a distinct fold in type A FTLD-TDP. Nature.

[36] Arseni D, Hasegawa M, Murzin AG, Kametani F, Arai M, Yoshida M (2022). Structure of pathological TDP-43 filaments from ALS with FTLD. Nature.

[37] Mahoney CJ, Beck J, Rohrer JD, Lashley T, Mok K, Shakespeare T (2012). Frontotemporal dementia with the C9ORF72 hexanucleotide repeat expansion: clinical, neuroanatomical and neuropathological features. Brain: J Neurol.

[38] Murray ME, DeJesus-Hernandez M, Rutherford NJ, Baker M, Duara R, Graff-Radford NR (2011). Clinical and neuropathologic heterogeneity of c9FTD/ALS associated with hexanucleotide repeat expansion in C9ORF72. Acta Neuropathol.

[39] Bigio EH, Weintraub S, Rademakers R, Baker M, Ahmadian SS, Rademaker A (2013). Frontotemporal lobar degeneration with TDP-43 proteinopathy and chromosome 9p repeat expansion in C9ORF72: clinicopathologic correlation. Neuropathology.

[40] Alafuzoff I, Pikkarainen M, Neumann M, Arzberger T, Al-Sarraj S, Bodi I (2015). Neuropathological assessments of the pathology in frontotemporal lobar degeneration with TDP43-positive inclusions: an inter-laboratory study by the BrainNet Europe consortium. J Neural Transm.

[41] Prudlo J, Konig J, Schuster C, Kasper E, Buttner A, Teipel S (2016). TDP-43 pathology and cognition in ALS: a prospective clinicopathologic correlation study. Neurology.

