# Supplementary figures and images for: Heterogeneity of cortical pTDP-43 inclusion morphologies in amyotrophic lateral sclerosis
Source: Acta Neuropathol Commun. 2023 Nov 13;11:180. doi: 10.1186/s40478-023-01670-2 (PMC10642010; doi:10.1186/s40478-023-01670-2)

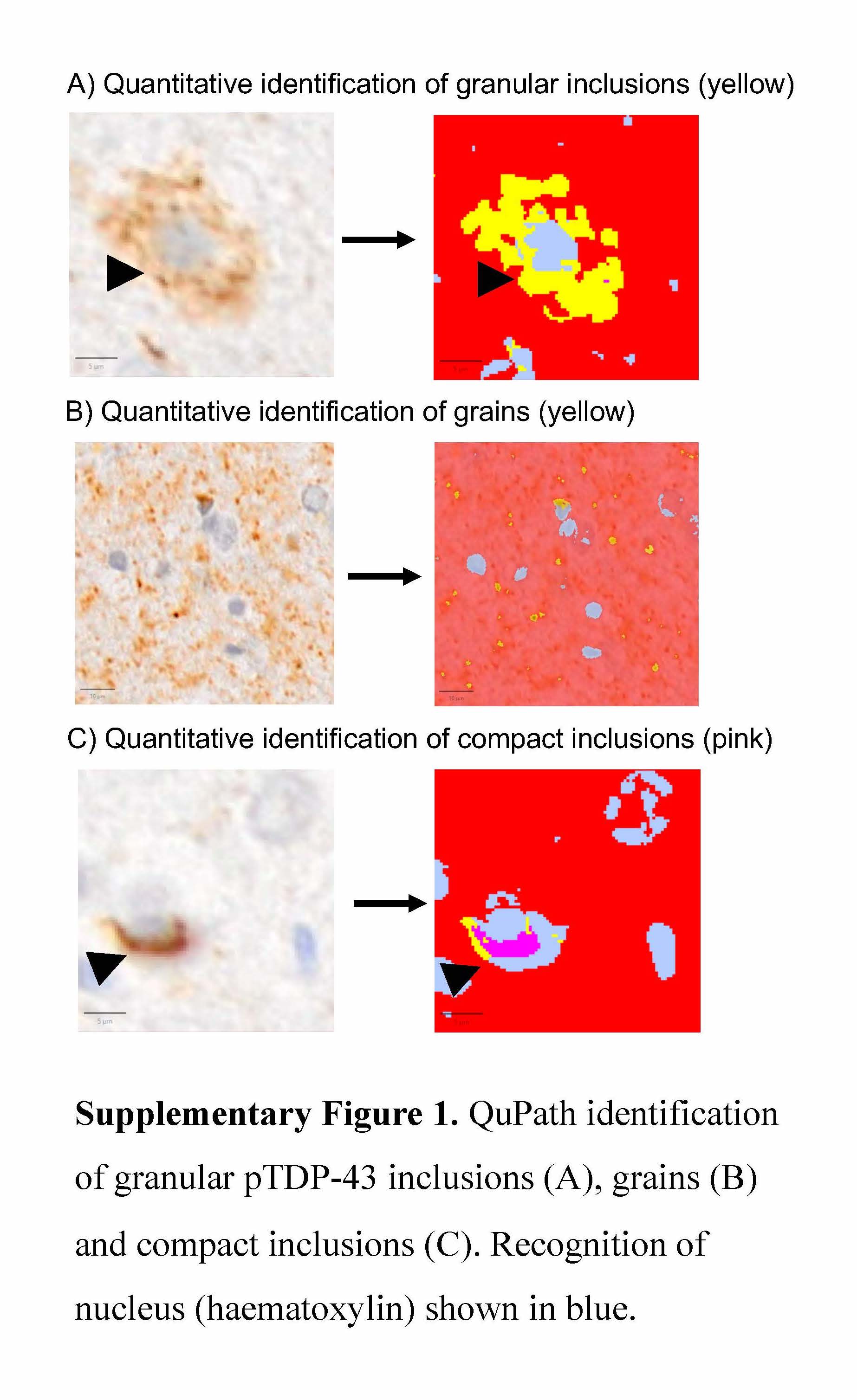

Supplement: Supplementary file 1 — Additional file 1: Supplementary Figure 1. [file 40478_2023_1670_MOESM1_ESM.jpg]
